# Disparities in deafness gene mutations between Han and Li ethnic newborns in Hainan, China: insights from a combined screening program

**DOI:** 10.1186/s12920-026-02392-9

**Published:** 2026-05-19

**Authors:** Xin Qi, Dan Wang, Shuo Liu, Bo Jiao, Xiujuan Tian, Pu Dai, Yu Su

**Affiliations:** 1 Department of Otolaryngology Head and Neck Surgery, Hainan Hospital of the Chinese People’s Liberation Army General Hospital, Sanya, 572013 China; 2 Hainan Clinical Research Center for Otolaryngology Head and Neck Diseases, Sanya, 572013 China; 3 Department of Otolaryngology Head and Neck Surgery, Chinese People’s Liberation Army General Hospital, Beijing, 100853 China; 4 National Clinical Research Center for Otolaryngologic Diseases, Beijing, 100853 China; 5 Shanghai Children’s Medical Center, Hainan Hospital, Affiliated to Shanghai Jiao Tong University School of Medicine(Sanya Maternal and Child Health Hospital), Sanya, 572000 China

**Keywords:** Hearing loss, Newborn screening, GJB2, Ethnic disparities, Hainan

## Abstract

**Objective:**

This study aimed to investigate the spectrum of deafness gene variants among newborns in Hainan Province, China. We also evaluated the effectiveness of a combined hearing and genetic screening approach in this region. Special attention was given to differences between the Han and Li populations, as well as the relationship between genetic variants and hearing follow-up outcomes.

**Methods:**

From October 2020 to June 2022, 2128 newborns in Hainan (1502 Han, 626 Li) received combined hearing and genetic screening. Hearing screening followed national standards. Genetic screening was carried out using targeted next-generation sequencing of *GJB2*,* SLC26A4*,* GJB3*,* and MT-RNR1*. Infants with detected variants or those referred from hearing screening received diagnostic audiological follow-up.

**Results:**

Among the 2128 newborns (1502 Han, 626 Li), 514 (24.15%) carried deafness gene variants. Variants in GJB2 were the most common, with a carrier rate of 22.51% (479/2128). The *GJB2* c.109G > A (p.V37I) variant was the hotspot variant, with an overall carrier rate of 20.91% (445/2128). This rate was significantly higher in the Li ethnic group (28.6%, 179/626) than in the Han group (17.7%, 266/1502; *P* < 0.01). Thirty-seven newborns carried biallelic pathogenic variants in *GJB2*. Nine newborns (0.42%) were diagnosed with hearing loss at three months, and all 9 had biallelic *GJB2* variants (7 homozygous c.109G > A, 1 compound heterozygote c.109G > A/299_300del, and 1 homozygous c.235del). Longitudinal follow-up of children with biallelic c.109G > A variants showed delayed-onset and progressive hearing loss, with confirmed cases increasing at ages 2 and 4. There are no significant differences in mean ABR thresholds or their standard deviations between Han and Li individuals with homozygous *GJB2* c.109G > A mutations at 4 years of age. For patients with biallelic c.109G > A variants, the current setting of ABR thresholds may lead to missed diagnosis.

**Conclusion:**

This study showed a high prevalence of deafness gene variants, especially *GJB2* c.109G > A, among newborns in Hainan, with clear ethnic differences. Combined screening was effective in identifying genetic hearing loss, including delayed-onset and progressive cases that could be missed by hearing screening alone. These findings support adding genetic screening to regional newborn screening programs and highlight the need for long-term monitoring of at-risk children.

## Introduction

Hearing loss is one of the most common disabling conditions and is also an important public health issue worldwide. Congenital hearing loss is considered a common birth defect in China, with a prevalence of 1–3 per 1,000 live births. About 50%–60% of cases have a genetic cause [1, 2]. Early diagnosis and treatment are important to reduce disability caused by deafness. The widespread use of universal newborn hearing screening has reduced the age of detection of congenital hearing loss from 24 to 30 months to around 3 months [3]. However, some types of genetic hearing loss may be delayed-onset or progressive and can pass the initial newborn hearing screening. In 2006, Morton [4] first suggested that genetic screening for deafness could increase the detection rate of prelingual deafness and help explain its causes. In 2007, Wang [5] suggested that adding molecular screening of deafness-susceptibility genes into the existing newborn hearing-screening system would be helpful in China. Deafness shows high genetic heterogeneity and involves many genes. Epidemiological studies have identified hotspot genes and variants in the Chinese deaf population. Based on these findings, we worked with Tsinghua University and CapitalBio Corporation to develop a deafness genetic detection chip. This chip targets 9 variants across 4 genes and has independent intellectual property rights, and it was certified by the China Food and Drug Administration (CFDA). The chip targets variants in *GJB2* (c.235del, c.299_300del, c.176del16/c.35del), *SLC26A4* (c.919–2 A > G, c.2168 A > G), mitochondrial DNA (m.1555 A > G, m.1494 C > T), and *GJB3* (c.538 C > T) [6–8].

Hainan Province in southern China has a unique ethnic composition and genetic structure, mainly due to its indigenous Li population. The Li ethnic group, a major indigenous minority in Hainan, has long maintained a high rate of endogamy, which may have led to a distinct genetic background. However, systematic research on the prevalence of deafness gene variants among newborns in Hainan is still limited, and combined hearing and genetic screening is not yet routinely carried out. Therefore, it is important to clarify the spectrum of deafness gene variants in Hainan newborns, examine differences in carrier rates between ethnic groups, explore genotype-phenotype relationships, and assess reproductive risks. These efforts are important for developing region- and ethnicity-specific strategies for deafness prevention and control.

## Methods

### Study subjects and workflow

**Fig. 1:**
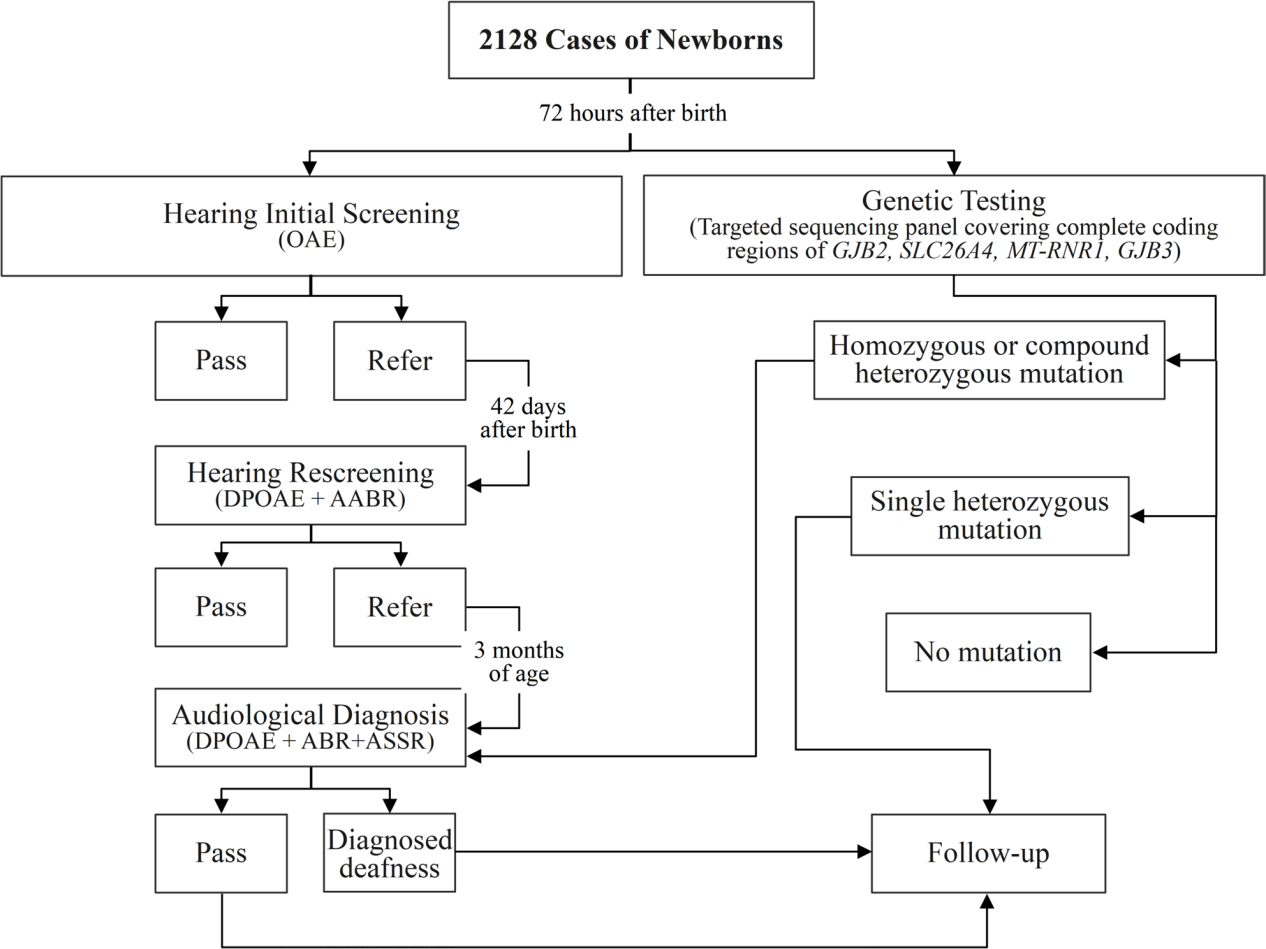
Workflow for combined newborn hearing and genetic screening. OAE: otoacoustive emmission; DPOAE: distortion product otoacoustive emmission; AABR: automated auditory brainstem response; ASSR: auditory steady-state responseto

A total of 2,128 newborns born at the Sanya Maternal and Child Health Hospital from October 2020 to June 2022 were included in this study. All participants received hearing and genetic screening at approximately 3 days after birth. Newborns with identified variants received genetic counseling, and confirmed cases received long-term audiological follow-up. The study protocol was approved by the Ethics Committee of Hainan Hospital of the Chinese People’s Liberation Army General Hospital (Approval No.: 2020 − 142). Written informed consent was obtained from the guardians of all participants. The screening workflow is shown in Fig. 1.

### Hearing screening

According to the Chinese Ministry of Health’s “Technical Specifications for Newborn Hearing Screening” [9], initial hearing screening was carried out using transient evoked otoacoustic emissions (TEOAE; EroScan, MAICO, Germany) within 72 h after birth in a room with ambient noise < 40 dB SPL. Infants who referred the initial screening received rescreening at 42 days of age. The rescreening used distortion product otoacoustic emissions (DPOAE) and automated auditory brainstem response (AABR), with the MB11 device (MAICO, Germany). Passing both DPOAE and AABR was considered a pass on rescreening. Infants who failed rescreening received diagnostic audiological evaluation, including DPOAE and ABR, at approximately 3 months of age. Hearing loss was defined as an air-conduction ABR threshold ≥ 35 dB nHL [10]. In cases of asymmetric loss, the better-hearing ear was used for analysis.

### Genetic screening

Capillary heel blood samples were collected onto standardized filter paper cards at 3 days of age according to the “Technical Specifications for Blood Collection for Neonatal Disease Screening.” Dried blood spots (DBS) were stored at 2–8 °C. Genomic DNA was extracted, and sequencing libraries were constructed. Targeted capture was then performed using the GenCap kit (MyGenostics, Beijing, China) to enrich the complete coding regions of *GJB2*,* SLC26A4*, and *GJB3*, as well as the *MT-RNR1* gene. Next generation sequencing was performed on an Illumina NextSeq 500 platform with an average depth of 300×. Variants identified through bioinformatics analysis were confirmed by Sanger sequencing.

### Bioinformatics analysis

Raw sequencing data were filtered to generate clean reads, which were then aligned to the human reference genome (GRCh37/hg19). Variant calling for single nucleotide polymorphisms and insertions or deletions was performed using GATK and VarScan, respectively. The identified variants were annotated using ANNOVAR against databases including dbSNP, 1000Genomes, and ESP6500. Only variants classified as pathogenic or likely pathogenic based on ACMG guidelines were retained. Variants were further filtered using the following criteria: a read depth of ≥ 5×, a variant allele frequency of ≥ 30%, and the exclusion of synonymous variants. In addition, variants with high population frequency (> 5%) in databases (1000g2015apr, ESP6500si, Inhouse, ExAC_ALL, ExAC_EAS) were excluded unless they had already been reported as pathogenic variants.

### Final determination of pathogenic variants

The above variant filtering criteria, including pathogenicity classification, population frequency < 5%, and non-synonymous variants, were applied for further screening. Based on the guidelines of the American College of Medical Genetics and Genomics (ACMG), the filtered candidate variants were analyzed together with the individual clinical phenotypes. This process led to the final selection of candidate pathogenic variants. The identified pathogenic variants were then validated by Sanger sequencing.

### Statistical analysis

All statistical analyses were performed using SPSS software (version 23.0). Categorical data are presented as frequencies and percentages. Differences in carrier rates between ethnic groups were analyzed using the Pearson chi-square test or Fisher’s exact test, as appropriate. The t-test was used to compare means between the two groups, and Levene’s test was used to compare standard deviations between the two groups. A two-sided P-value < 0.05 was considered statistically significant.

## Results

### Results of genetic screening

This study included 2,128 newborns, including 1,135 males and 993 females. Among them, 1,502 were Han and 626 were Li. A total of 514 newborns (24.15%) were found to carry deafness gene variants. Specifically, 479 (22.51%), 26 (1.22%), 12 (0.56%), and 2 (0.094%) carried variants in *GJB2*, *SLC26A4*, *MTRNR-1*, and *GJB3*, respectively. Within the Li group, the carrier rates for *GJB2* and *SLC26A4* were 28.75% (180/626) and 0.32% (2/626). In the Han group, the rates were 19.91% (299/1502) for *GJB2* and 1.60% (24/1502) for *SLC26A4*. Variants in *GJB2* were the most common. Thirty-seven neonates carried biallelic *GJB2* variants and were confirmed or considered at high risk for hereditary hearing loss. Detailed data on variant carriers are shown in Table 1.

**Table 1:** Pathogenic variant or Likely Pathogenic Variant Spectrum and Carrier Rate of Hereditary Deafness-Related Genes in 2128 Newborns

Variants type	Variants site	Cases(*n*)	Ethnic Distribution (Li/Han)	Percentage (%,*N* = 2128)
*GJB2* biallelic variant	c.109G > A homozygous	29	16/13	1.36
	c.109G > A /c.235del	4	1/3	0.19
	c.109G > A /c.299_300del	3	0/3	0.14
	c.235del homozygous	1	0/1	0.047
*GJB2 s*ingle heterozygous variant	c.109G > A	404	157/247	18.98
	c.235del	23	2/21	1.08
	c.299_300del	5	1/4	0.23
	c.416G > A	1	0/1	0.047
	c.35del	1	0/1	0.047
	c.230G > A	1	1/0	0.047
	c.35dup	1	0/1	0.047
	c.511_512ins	1	0/1	0.047
*SLC26A4 s*ingle heterozygous variant	c.919–2 A > G	16	0/16	0.75
	c.2086 C > T	4	0/4	0.19
	c.1229 C > T	1	0/1	0.047
*GJB2* and *SLC26A4* *s*ingle heterozygous variant	*GJB2* c.109G > A *SLC26A4* c.919–2 A > G	4	2/2	0.19
	*GJB2* c.109G > A *SLC26A4* c.2086 C > T	1	0/1	0.047
*GJB3 s*ingle heterozygous variant	c.497 A > G	2	0/2	0.094
Mitochondrial gene variant	m.1555 A > G	9	2/7	0.42
	m.1494 C > T	3	1/2	0.14
	Total	514	183/331	24.15

**Fig. 2:**
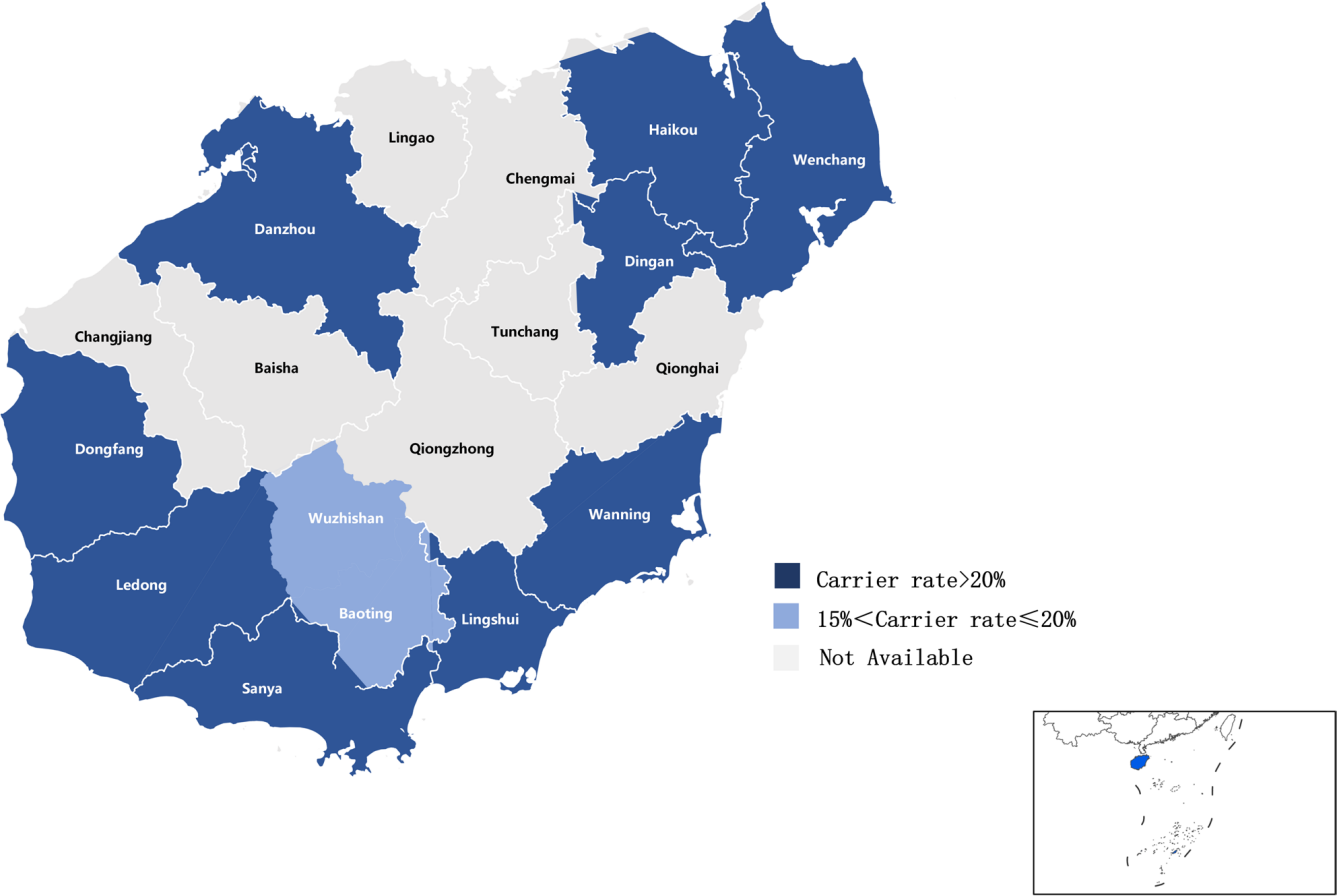
The GJB2 variant carrier status across regions with more than 30 sampled newborns

The *GJB2* c.109G > A variant was the hotspot variant in this study population. A total of 445 individuals carried this variant, giving an overall carrier rate of 20.91%. As shown in Table 2, the carrier rate was 17.7% in the Han group and 28.6% in the Li group. This difference was statistically significant (χ² = 31.65, *P* < 0.01). For the *GJB2* c.235del variant, the carrier rates were 1.66% in the Han group and 0.32% in the Li group, which was also statistically significant (χ² = 4.68, *P* = 0.029). The distribution of *GJB2* variant carriers across regions with more than 30 sampled newborns is shown in Fig. 2. Overall, Hainan Province showed a high carrier rate of *GJB2* variants.

**Table 2:** Carrier Frequencies of Two Common *GJB2* Variants

Ethnicity	Carrier of c.109G > A	Non-carrier of c.109G > A	Carrier of c.235del	Non-carrier of c.235del
Han	266	1236	25	1477
Li	179	447	3	623
Total	445	1683	28	2100
Statistical value	χ² = 31.65		χ² = 4.68	
* P* value	*P* < 0.01		*P* = 0.029	

### Results of combined newborn hearing and genetic screening

The combined hearing and genetic screening results are shown in Table 3. A total of 1,996 newborns (93.80%) passed the initial hearing screening, and 2,070 (97.27%) passed the rescreening. Fifty-eight newborns (2.73%) were referred for rescreening. Among these, nine (0.42%) were diagnosed with hearing loss at three months of age. Seven carried biallelic *GJB2* c.109G > A variants and were diagnosed with mild-to-moderate sensorineural hearing loss. One infant with a homozygous *GJB2* c.235delC variant had bilateral severe sensorineural hearing loss and later received cochlear implantation. The positive rate of deafness gene variants was 31.06% among infants who failed the initial screening and 17.24% among those who failed the rescreening. Etiological diagnosis and hearing phenotype analysis of the 58 infants who failed rescreening are shown in Table 4.

**Table 3:** Analysis of differences between hearing screening and genetic screening results (n, %)

Screening Process & Result	Variants Negative (*n* = 1614)	Variants Positive (*n* = 514)	χ² Value	*P* value
Initial Screening				
Pass	1523 (94.36%)	473 (92.02%)	3.67	0.056
Refer	91 (5.64%)	41 (7.98%)		
Rescreening (in initial refers)	*n* = 91	*n* = 41		
Pass	43 (47.25%)	31 (75.61%)	8.54	0.003
Refer	48 (52.75%)	10 (24.39%)		

**Table 4:** Etiological diagnosis and hearing phenotype analysis of newborns who failed hearing rescreening (*n* = 58)

Etiology	Cases	Genotype (Number of Cases)	Audiological Phenotype			
			Tympanometry	DPOAE	AABR(right)	AABR(left)
Confirmed Genetic Deafness	9	c.109G > A Hom (7)	Type A	Bilateral Fail	35-40dB nHL	35-45dB nHL
		c.235del Hom(1)	Type A	Bilateral Fail	80dBnHL	80dB nHL
		c.299_300del/c.109G > A(1)	Type A	Bilateral Fail	35dBnHL	50dB nHL
Uncertain Significance	1	c.511_512ins single Heterozygous(1)	Type A	Bilateral pass	25dB nHL	20dB nHL
Non-genetic/Transient	48	No pathogenic variant detected(48)	Type A	Bilateral pass	20-25dB nHL	20-25dB nHL

**Fig. 3:**
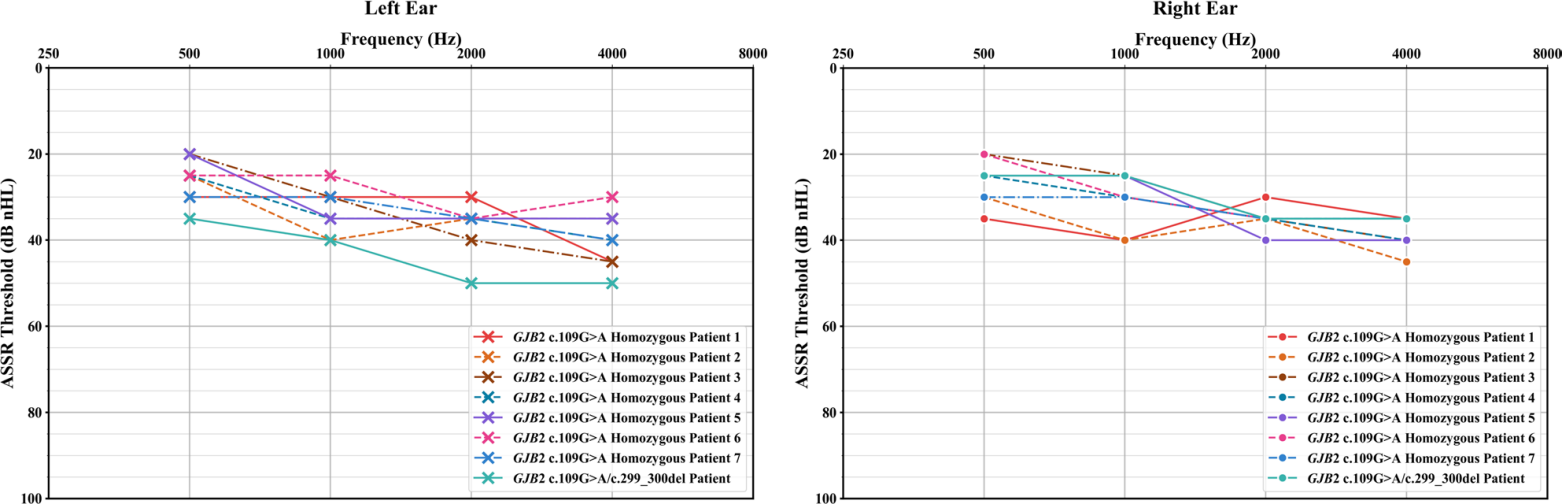
Results of ASSR in both ears of a child with biallelic GJB2 c.109G>A variants

Audiological follow-up of 10 children who failed rescreening confirmed bilateral hearing loss in 9 cases (90.0%) and normal hearing in 1 case (10.0%). ASSR results of 7 cases with homozygous *GJB2* c.109G > A variants are shown in Fig. 3. All seven showed bilateral and roughly symmetric sensorineural hearing loss, with gently sloping or flat hearing curves. One infant with compound heterozygous *GJB2* c.109G > A / c.299_300del variants showed asymmetric hearing loss, which was more severe in the left ear.

### Hearing follow-up results in newborns with biallelic *GJB2* variants

**Fig. 4:**
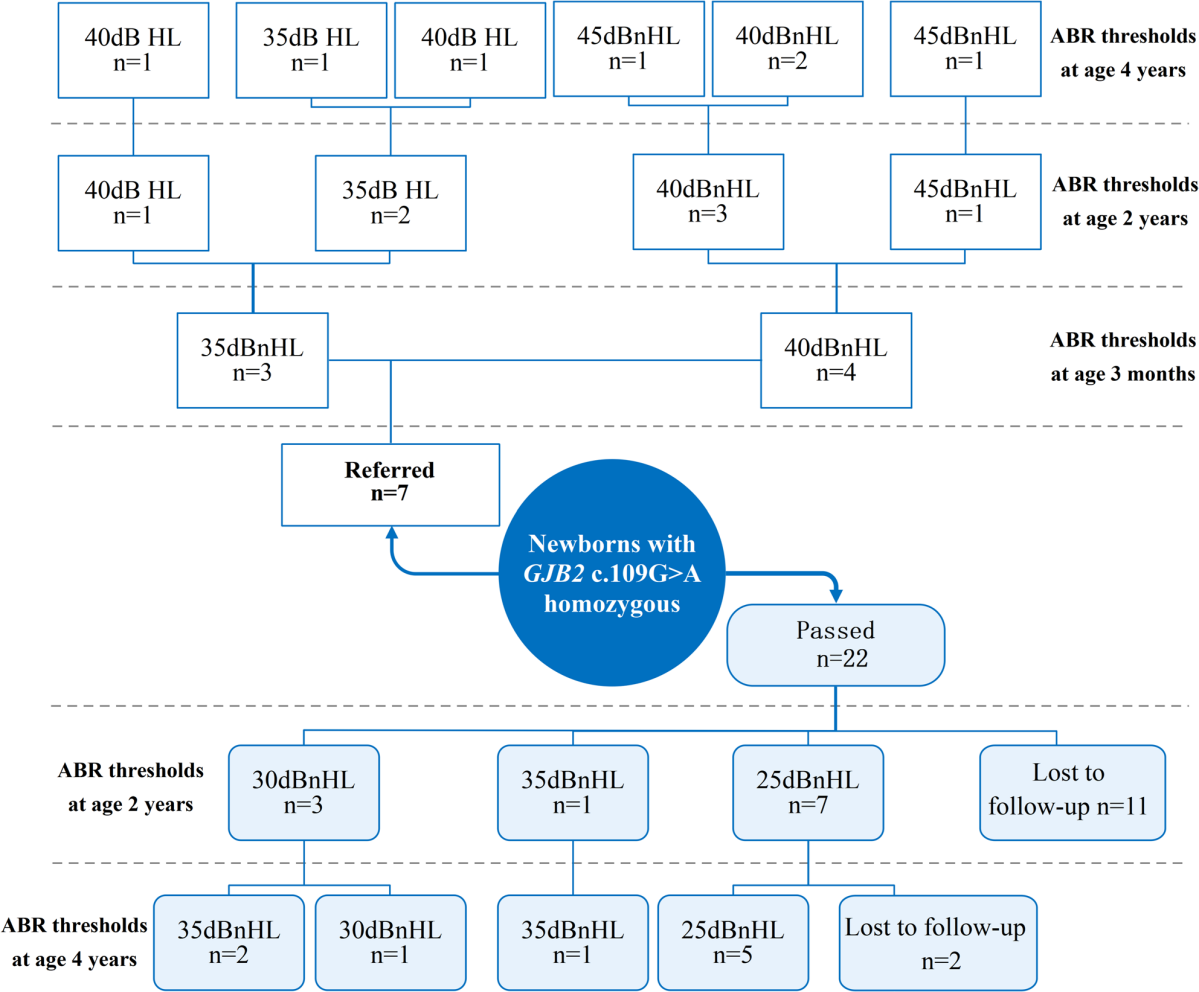
Hearing Follow-up Results in Newborns with GJB2 c.109G>A Homozygous Variants

**Fig. 5:**
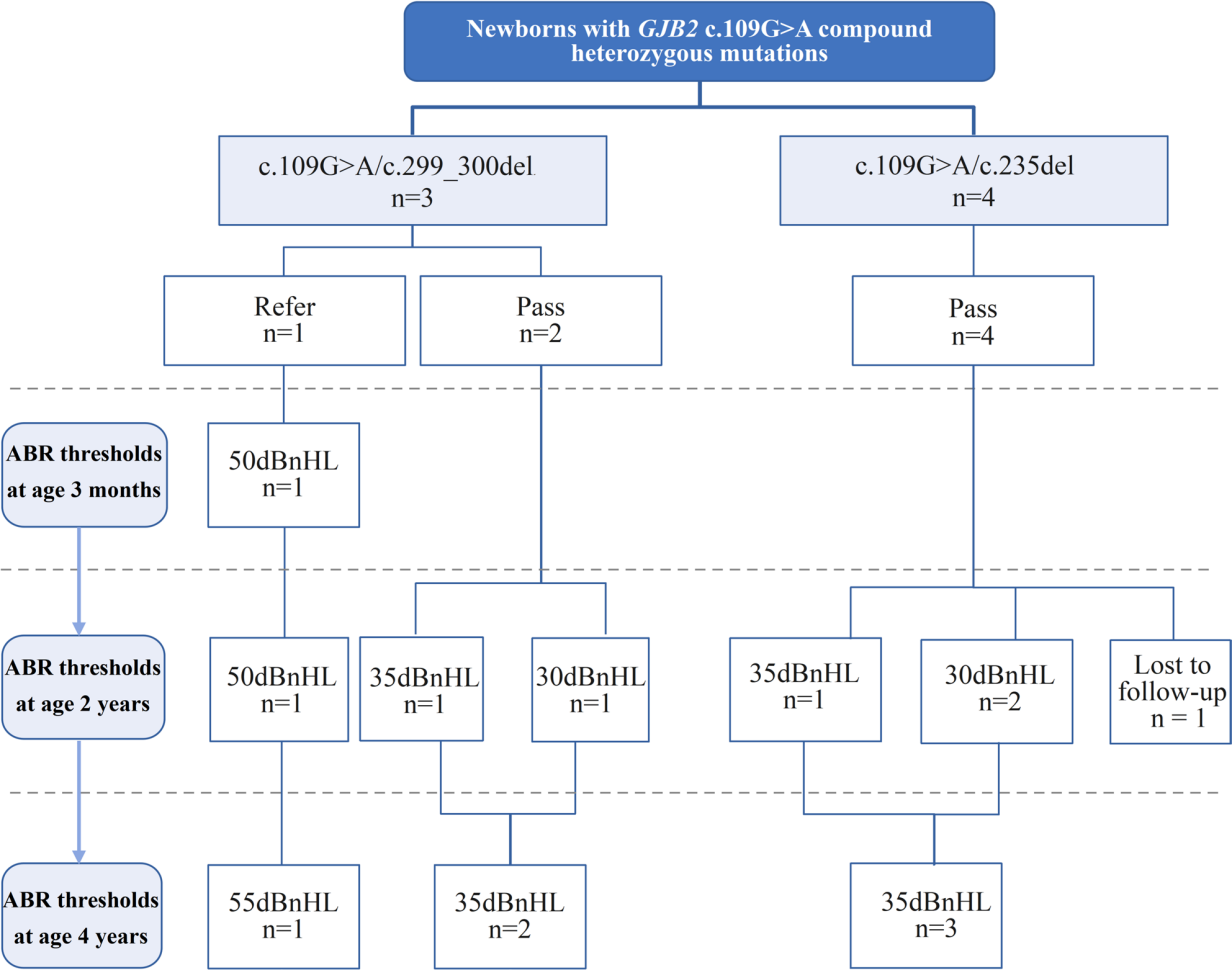
Hearing Follow-up Results in Newborns with GJB2 c.109G>A Compound Heterozygous Variants

**Fig. 6:**
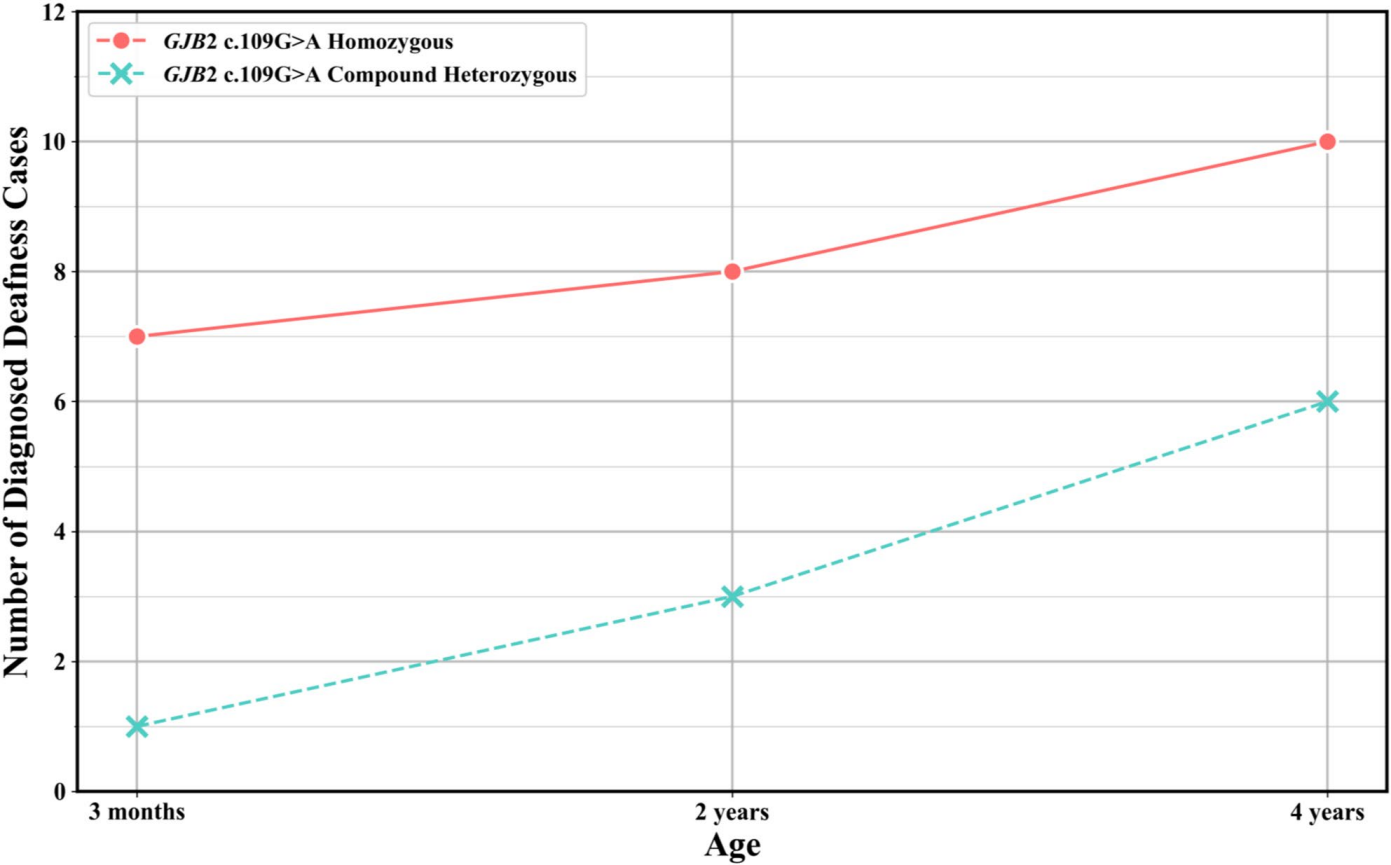
Trend in confirmed deafness cases over time

**Fig. 7:**
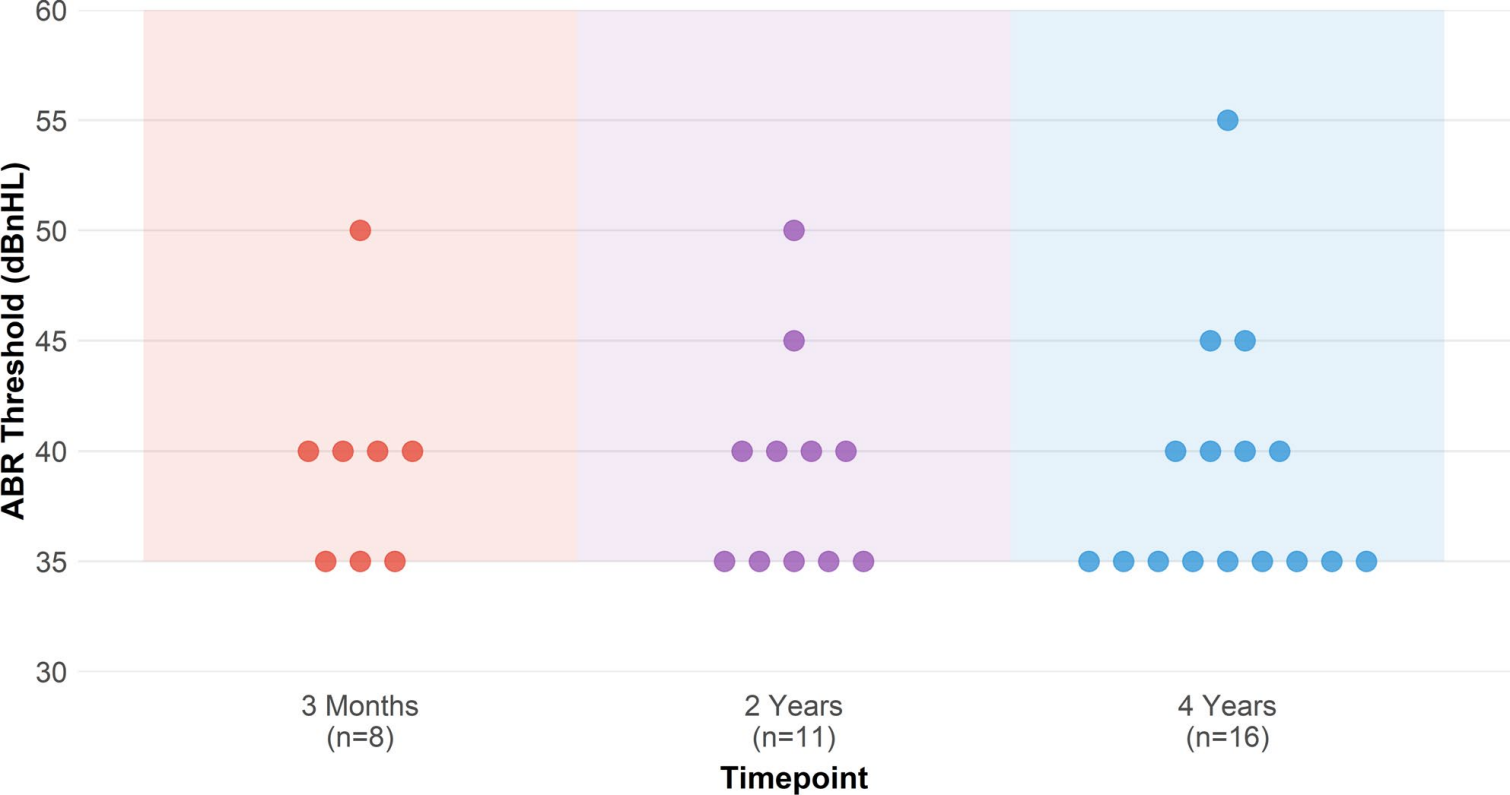
ABR Thresholds in Newborns with GJB2 c.109G>A Biallelic Variants Confirmed with Hearing Loss

**Fig. 8:**
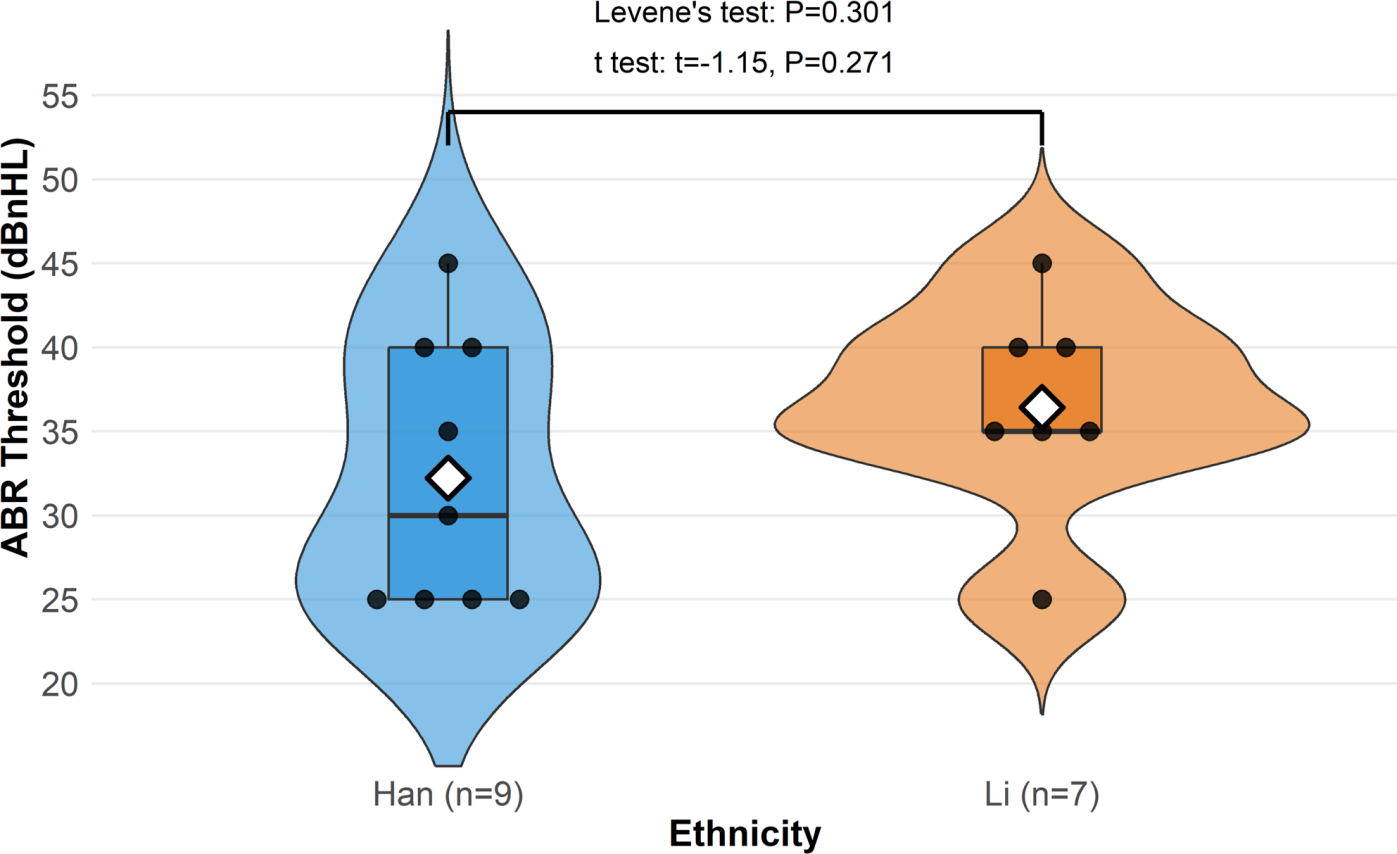
Hearing Follow-up Results in Newborns with GJB2 c.109G>A Compound Homozygous Variants

As shown in Figs. 4 and 5, guardians of newborns with biallelic GJB2 c.109G > A variants were contacted by phone and asked to return to the Department of Otolaryngology-Head and Neck Surgery, Hainan Hospital of the Chinese People’s Liberation Army General Hospital for diagnostic ABR testing when the children reached 2 and 4 years of age. At the 2-year follow-up, 24 newborns participated, giving a follow-up rate of 66.67%. At the 4-year follow-up, 22 children participated, with a follow-up rate of 61.11%. Fourteen families declined follow-up. Among newborns with the c.109G > A homozygous variant, 8 cases were diagnosed with hearing loss at 2 years of age and 10 cases at 4 years of age. Among those with the c.109G > A compound heterozygous variant, 3 cases were diagnosed with hearing loss at 2 years of age and 6 cases at 4 years of age. Among the 36 newborns with biallelic *GJB2* c.109G > A variants, 16 (44.4%) were confirmed to have hearing loss by diagnostic ABR at 4 years of age. Fourteen newborns were lost to follow-up, and the remaining 6 maintained normal hearing thresholds at their last visit. As shown in Fig. 6, the follow-up results indicate that biallelic *GJB2* variants show delayed-onset and progressive hearing loss, and the number of confirmed cases increased over time. Figure 7 shows ABR thresholds at 3 months, 2 years, and 4 years in newborns with biallelic *GJB2* c.109G > A variants. The number of individuals with thresholds ≥ 35 dB nHL increased from 3 to 9 over time. A worsening trend in hearing phenotype was also seen among those already diagnosed with hearing loss. Figure 8 shows no significant differences in mean ABR thresholds or their standard deviations between Han and Li individuals with homozygous *GJB2* c.109G > A variants at 4 years of age. As shown in Table 5, hearing screening alone identified 9 cases (0.42%) of confirmed hearing loss or drug-induced susceptibility. Both genetic screening alone and the combined strategy identified 29 cases (1.36%), showing that genetic screening contributed to all additional cases beyond those identified by hearing screening.

**Table 5:** Detection efficacy of combined screening for hearing loss in this study (*N* = 2128)

	Number of confirmed cases with hearing loss or susceptibility to drug-induced hearing loss	Rate of confirmed cases with hearing loss or susceptibility to drug-induced hearing loss
Hearing screening	9	0.42%
Genetic screening	29	1.36%
Combined screening	29	1.36%

In this cohort, individuals who did not complete follow-up or were not diagnosed by the end of the follow-up period were excluded from the efficacy calculation

## Discussion

### Value of combined newborn hearing and genetic screening

Universal newborn hearing screening has been widely used worldwide and plays an important role in the early detection, diagnosis, and intervention of hearing loss. It has reduced the average age of confirmed hearing impairment from 24 to 30 months to about 3 months. However, delayed-onset or drug-induced hearing loss shows the limitations of conventional hearing screening. Adding genetic screening for deafness can help address these gaps [4, 5]. In 2006, Morton [4] first suggested that genetic screening could improve the detection rate of prelingual deafness and help explain its causes. In 2007, Wang Qiuju [5] proposed in China that molecular screening for susceptibility genes should be integrated into the widely used newborn hearing screening program. In their follow-up study of 12,778 newborns, genetic screening identified 13% more infants with hearing impairment than hearing screening alone. It also identified 2,638 newborns (0.23% of the total) who were susceptible to drug-induced hearing loss, which cannot be detected by hearing screening alone. These findings show that adding genetic screening can improve the effectiveness of newborn hearing screening programs. In this study, the combined screening did not perform better than genetic screening alone. This suggests that all confirmed cases or susceptible individuals were identified by genetic testing. This supports adding genetic screening into newborn hearing screening programs, especially for detecting delayed-onset hearing loss and drug-induced susceptibility, which cannot be identified by physiological tests alone. It should be noted that no case of confirmed hearing loss with negative genetic screening was found in this study. This may be related to the relatively small sample size, since non-genetic causes of hearing loss, such as perinatal infections or ototoxic drugs, are expected to be rare in the general newborn population. Larger studies are needed to detect such cases and to better evaluate how hearing and genetic screening complement each other. Long-term hearing follow-up showed increased penetrance in individuals with biallelic c.109G > A variants. This helped reduce missed cases in high-risk infants and showed a complementary effect between hearing and genetic screening. In the past, *GJB2* c.109G > A did not receive much attention due to limited evidence on its pathogenicity. Since the 2019 ACMG guidelines classified the c.109G > A (p.V37I) variant as pathogenic [10], more studies in China have focused on this variant. It shows a pattern of higher frequency in southern China than in the north, and biallelic variants are often linked with delayed-onset and progressive hearing loss [12–16]. Future screening strategies should consider including c.109G > A as an important target and pay attention to hearing changes in adolescents. In addition, this study found a considerable number of carriers with single heterozygous pathogenic variants. Although these newborns passed hearing screening, they may benefit from genetic counseling. Their future reproductive decisions and the risk of deafness in their offspring should be considered.

### Newborn deafness screening in the hainan region

According to demographic data from Hainan (hainan.gov.cn), by the end of 2022, the Han population accounted for 82.32% of the total population, the Li population accounted for 15.67%, and other minority groups accounted for 2.01%. Nationwide, the Li population is about 1.46 million, with around 1.26 million living in Hainan. Despite this large Li population, there are still limited systematic reports on combined hearing and genetic screening in Hainan, especially for the Li group. Huang et al. [11] conducted the first molecular study on 299 patients with non-syndromic hearing loss in Hainan. They found that the most common variant, *GJB2* c.109G > A, had an allele frequency of 15.05%, which is higher than in mainland China. In this study, genetic screening of 2,128 newborns in Hainan provided a clearer picture of the deafness gene variant spectrum in both Han and Li populations in central and southern Hainan. The *GJB2* c.109G > A variant was again identified as a hotspot, with a carrier rate of 20.91%. Compared with data from the Molecular Diagnosis Center for Deafness at the Chinese PLA General Hospital, this frequency is about 2.6 times the national average (about 7.83%) and also higher than the reported 12% in southern China. The proportion of Li newborns carrying this variant was significantly higher than that of Han newborns. This is consistent with findings from Zhao’s studies [13, 14] on the Li population in central and northern Hainan. Xu et al. [15] studied 585 newborns of the Miao ethnicity in central Hainan and found that 36.1% carried deafness gene variants. The carrier rates of GJB2 and SLC26A4 were 27.35% and 4.79%, and c.109G > A remained the most common variant. Taken together, current studies suggest that c.109G > A is widely distributed across different regions and ethnic groups in Hainan rather than being limited to specific areas. It is likely the most common deafness-related variant in this region. The higher carrier rate in the Li population may be related to long-term endogamy, but this study did not directly examine the genetic mechanisms behind this pattern. Therefore, this explanation remains tentative and needs further study.

### Clinical management of biallelic *GJB2* c.109G > A variants

For biallelic c.109G > A variants, reported phenotypes vary from normal hearing to mild or moderate hearing loss [17]. Long-term follow-up studies have shown that children with biallelic *GJB2* c.109G > A variants can develop delayed-onset and progressive hearing loss [18]. These findings provide useful information for improving regional strategies for deafness prevention and control. One key issue is the choice of ABR threshold. According to Zheng et al. [18], 34.0% of individuals with biallelic c.109G > A variants had ABR thresholds in the 20–35 dB nHL borderline range. If clinical practice uses “ABR threshold ≥ 35 dB nHL” as the cutoff for normal hearing, individuals in this range may be missed. Li [19] also reported that some infants with ABR thresholds ≤ 35 dB nHL, who were considered normal in hearing screening, were carriers of *GJB2* c.109G > A variants. In this study, the number of individuals with thresholds ≥ 35 dB nHL increased from 3 to 9 over time. A worsening trend in hearing phenotype was also seen among those already diagnosed with hearing loss. These suggest that the commonly used threshold may not be suitable for all populations [20]. A more appropriate threshold may need to consider regional characteristics, cost-effectiveness, and available resources in newborn screening programs. Follow-up in this study identified the risk of delayed-onset and progressive hearing loss in newborns with biallelic *GJB2* variants. Among 36 newborns with biallelic *GJB2* c.109G > A variants, 16 were confirmed to have hearing loss by ABR at 4 years of age. Fourteen were lost to follow-up, and the remaining 6 had normal hearing at their last visit. This loss to follow-up may be related to guardians believing that follow-up was unnecessary after normal screening results and behavior. Some infants with biallelic c.109G > A variants who initially had normal or mild hearing showed clear threshold increases later. Five showed hearing decline at age 2 and eight at age 4. The loss of 14 cases may affect the estimation of hearing loss progression and suggests that some guardians may not fully understand the genotype-phenotype relationship. This is in line with previous studies suggesting that biallelic *GJB2* c.109G > A variants are associated with progressive hearing loss. However, the rate of decline over time is still unclear. Chen et al. [21] reported an average annual decline of about 0.4 dB in a large sample of carriers. Some studies suggest that compound heterozygous variants may lead to more severe hearing loss than homozygous variants, especially in children [18, 22], while other studies in adults did not find clear differences [23, 24]. We also compared hearing outcomes between Li (*n* = 9) and Han (*n* = 7) children with homozygous *GJB2* c.109G > A variants at 4 years of age. No significant differences were found in hearing loss severity or ABR variability, although the sample size was small. Whether the higher genetic homogeneity of the Li population affects phenotypic variation needs to be studied in larger cohorts. In general, children identified with biallelic *GJB2* c.109G > A variants should receive long-term hearing follow-up, even if their initial hearing is normal. This is consistent with current recommendations for managing at-risk children.

### Limitation

This study has several limitations. As a single-center study, the findings may not fully represent the overall population of Hainan. The observed difference in the carrier rate of *GJB2* c.109G > A between ethnic groups is statistically significant but mainly descriptive. Loss to follow-up is another limitation and may introduce bias when assessing hearing loss progression. If those lost to follow-up had milder or no hearing loss, the actual progression rate may be lower than observed. Future studies should focus on improving follow-up compliance.

## Conclusions

This study shows a high prevalence and a distinct pattern of deafness gene variants among newborns in Hainan. The *GJB2* c.109G > A variant is a major hotspot and shows clear differences in carrier rates between Han and Li populations. Combined hearing and genetic screening is effective in identifying genetic hearing loss, especially delayed-onset and progressive cases that may be missed by hearing screening alone. These findings support adding genetic screening to regional newborn screening programs and highlight the importance of long-term audiological follow-up for at-risk infants. This will help in developing more targeted strategies for deafness prevention in Hainan.

## Data Availability

The data presented in this study are available on request from the corresponding author. The data are not publicly available due to privacy and ethical restrictions.
